# CT-Based Three-Dimensional Volumetric Analysis of Posterior and Lateral Malleolar Fragments in SER-Type Trimalleolar Ankle Fractures: Correlation and Reproducibility Study

**DOI:** 10.3390/tomography12070101

**Published:** 2026-07-04

**Authors:** Ruhat Ünlü, Barış Yılmaz, Hasan Emirhan Usta, Hamit Çağlayan Kahraman, Gülşah Yıldırım, Celaleddin Bildik

**Affiliations:** 1 Department of Orthopedics and Traumatology, Istanbul Atlas University, Istanbul 34408, Turkey; 2 Department of Orthopedics and Traumatology, Fatih Sultan Mehmet Training and Research Hospital, University of Health Sciences, Istanbul 34752, Turkey; drbyilmaz@yahoo.com (B.Y.); drhcaglayan@gmail.com (H.Ç.K.); 3 Department of Orthopedics and Traumatology, Gebze Fatih State Hospital, Kocaeli 41400, Turkey; mdemirhanusta@gmail.com; 4 Department of Radiology, University of Rumeli, Silivri 34570, Turkey; dr.gulsah.yildirim@gmail.com; 5 Private Clinic, OrthoRen Clinic, Istanbul 34750, Turkey; drcbildik@hotmail.com

**Keywords:** trimalleolar ankle fracture, posterior malleolus, lateral malleolus, computed tomography, three-dimensional imaging, volumetric segmentation, fracture morphology, quantitative imaging, reproducibility, intraclass correlation coefficient

## Abstract

Background: Posterior malleolar fractures are commonly assessed using two-dimensional measurements and morphology-based classification systems. However, ankle fracture morphology is inherently three-dimensional, and the reproducibility of CT-based volumetric segmentation for malleolar fracture fragments has not been sufficiently established. Objectives: This study aimed to evaluate the relationship between lateral and posterior malleolar fragment volumes in homogeneous supination–external rotation (SER)-type trimalleolar ankle fractures and to assess the intraobserver and interobserver reproducibility of a manual CT-based three-dimensional volumetric segmentation workflow. Methods: This retrospective musculoskeletal imaging study included 71 patients with SER-type trimalleolar ankle fractures who underwent preoperative computed tomography (CT). Posterior and lateral malleolar fracture fragments were segmented on thin-slice axial CT images using a standardized manual contour-based slice-by-slice workflow. Fragment volumes were calculated using dedicated volumetric imaging software. The association between lateral and posterior malleolar fragment volumes was assessed using Spearman correlation and multivariable linear regression analyses. Measurement reproducibility was evaluated using intraclass correlation coefficients for absolute agreement [ICC(A,1)] and Bland–Altman analyses. Results: The median lateral and posterior malleolar fragment volumes were 8.63 cm^3^ (interquartile range [IQR], 7.18–10.71) and 2.64 cm^3^ (IQR, 1.88–4.24), respectively. A weak but statistically significant positive correlation was observed between lateral and posterior malleolar fragment volumes (Spearman rho = 0.313, *p* = 0.008). In multivariable linear regression analysis, lateral malleolar fragment volume remained independently associated with posterior malleolar fragment volume after adjustment for age, sex, and body mass index (B = 0.316, standardized β = 0.39, *p* = 0.002). Intraobserver and interobserver reproducibility were excellent for all volumetric measurements, with ICC(A,1) values ranging from 0.996 to 0.999. Bland–Altman analyses demonstrated low mean bias and narrow limits of agreement across all comparisons. Geometric agreement was also excellent, with Dice similarity coefficient values ranging from 0.93 to 0.96 across intraobserver and interobserver segmentation comparisons.

## Simple Summary

Trimalleolar ankle fractures are commonly evaluated using computed tomography, but the size and morphology of fracture fragments are still often assessed with two-dimensional measurements. However, the shape of posterior and lateral malleolar fragments is three-dimensional and may not be fully represented by linear measurements alone. In this study, we used thin-slice computed tomography images to manually segment and calculate the volumes of posterior and lateral malleolar fracture fragments in patients with supination–external rotation type trimalleolar ankle fractures. We found a weak but statistically significant relationship between lateral and posterior fragment volumes. More importantly, the manual three-dimensional segmentation workflow showed excellent reproducibility across repeated measurements and among observers. These findings suggest that CT-based volumetric assessment may provide a reliable quantitative imaging method for describing fracture morphology. However, this approach should currently be considered complementary to standard clinical and radiological assessment, and further studies are needed to determine its relationship with surgical decision-making and patient outcomes.

## 1. Introduction

Trimalleolar ankle fractures are complex rotational injuries involving the lateral, medial, and posterior malleoli. Among these components, posterior malleolar involvement has received increasing attention because of its association with articular surface integrity, ankle stability, syndesmotic function, and overall fracture morphology [1,2]. Accordingly, computed tomography (CT) has become an important imaging modality for evaluating posterior malleolar fractures and characterizing fracture configuration beyond conventional radiography [3]. Despite the increasing use of CT, posterior malleolar fractures are still commonly assessed using two-dimensional (2D) measurements and morphology-based classification systems. Fragment size estimations, axial-slice-based linear measurements, and CT-based classifications, including those proposed by Haraguchi and Bartoníček/Rammelt, are widely used in clinical practice [1,2]. However, ankle fracture morphology is inherently three-dimensional (3D), and the complex geometry of malleolar fracture fragments may not be fully represented by planar measurements alone. This limitation is particularly relevant for posterior malleolar fragments, which often demonstrate oblique orientation, irregular fracture surfaces, articular extension, and multifragmentary morphology. Therefore, conventional 2D assessment may provide only a partial representation of the true spatial characteristics of the fracture. In recent years, quantitative imaging approaches have gained increasing relevance in musculoskeletal radiology. CT-based 3D volumetric assessment may provide a more comprehensive and objective method for quantifying fracture morphology. Unlike linear measurements, volumetric analysis allows fracture fragments to be assessed as continuous 3D structures and may better reflect the spatial complexity of irregular osseous fragments. However, studies evaluating the volumetric characteristics of malleolar fracture fragments remain limited. Moreover, before CT-based volumetry can be considered for broader clinical or research use, the reproducibility of the segmentation workflow must be established. This is particularly important for manual volumetric segmentation, where measurements may be influenced by image quality, fracture complexity, software tools, anatomical interpretation, and observer-dependent contour delineation [4,5,6]. Current literature has largely focused on posterior malleolar morphology as an isolated component, whereas the 3D relationship between lateral and posterior malleolar fragments has been investigated only to a limited extent. Lauge–Hansen supination–external rotation (SER)-type trimalleolar fractures may provide a relatively homogeneous model for quantitative fracture morphology assessment because the lateral and posterior malleolar components arise within a shared rotational injury mechanism [7]. Evaluating these fragments together may therefore provide additional insight into the spatial characteristics of SER-type trimalleolar fractures beyond conventional fragment-size measurements or morphology-based classifications. The primary aim of the present study was to evaluate the relationship between lateral and posterior malleolar fragment volumes in homogeneous SER-type trimalleolar ankle fractures using CT-based 3D volumetry. The secondary aim was to assess the intraobserver and interobserver reproducibility of a standardized manual CT-based volumetric segmentation workflow. We hypothesized that CT-based volumetric analysis would provide a reproducible quantitative imaging method for assessing malleolar fracture morphology and that lateral and posterior malleolar fragment volumes would demonstrate a measurable 3D association in SER-type trimalleolar fractures.

## 2. Materials and Methods

### 2.1. Study Design and Ethical Approval

This retrospective observational musculoskeletal imaging study was designed to evaluate the relationship between lateral and posterior malleolar fragment volumes in homogeneous Lauge–Hansen SER-type trimalleolar ankle fractures and to assess the reproducibility of a standardized CT-based volumetric segmentation workflow. Ethical approval was obtained from the Institutional Review Board of Fatih Sultan Mehmet Training and Research Hospital before study initiation (approval date: 10 December 2020; approval number: FSMEAH-KAEK-2020/122). The study was conducted in accordance with the principles of the Declaration of Helsinki. All clinical and imaging data were anonymized before analysis.

### 2.2. Patient Population

The institutional imaging archive was retrospectively reviewed to identify patients treated for trimalleolar ankle fractures between January 2017 and November 2020. Initially, 162 patients with trimalleolar ankle fractures were identified. The inclusion criteria were as follows: age 18 years or older, Lauge–Hansen SER-type trimalleolar ankle fracture, availability of preoperative CT imaging, and availability of 0.625 mm thin-slice axial CT reconstructions generated directly from the original raw CT acquisition data. The exclusion criteria were previous ankle surgery, pathological fracture, fracture mechanisms other than SER, incomplete CT datasets, unavailability of thin-slice CT reconstruction data, and severe motion or metal artifacts resulting in non-diagnostic image quality. Because the primary objective of this study was to evaluate volumetric relationships and segmentation reproducibility within a homogeneous fracture pattern, the study population was restricted to Lauge–Hansen SER-type trimalleolar ankle fractures. Of the initial cohort, 52 patients were excluded because their fracture mechanisms were not SER. Additional exclusions were made because preoperative CT images were unavailable in 21 patients, thin-slice reconstruction data were insufficient in 11 patients, and severe imaging artifacts prevented reliable evaluation in 7 patients. Consequently, 71 patients were included in the final analysis. Fracture mechanism was classified according to the Lauge–Hansen classification using preoperative plain radiographs and CT images. In our institution, standard ankle trauma assessment routinely includes preoperative plain radiographs before CT examination. Therefore, plain radiographs were available for all patients who met the CT-based inclusion criteria and were not listed as a separate eligibility criterion. All cases were independently reviewed by an orthopedic surgeon experienced in ankle trauma and a radiologist experienced in musculoskeletal imaging. Disagreements regarding fracture classification were resolved by consensus.

### 2.3. CT Imaging Protocol

All CT examinations were performed using a 128-slice multidetector CT scanner (Optima 660 SE, GE Healthcare, Chicago, IL, USA) according to the institutional ankle trauma imaging protocol. The scanning parameters included a tube voltage of 120 kVp and tube current of 300 mA. Axial images were initially reconstructed with a slice thickness of 2.5 mm for routine clinical evaluation. In addition, true thin-slice axial images with a reconstructed slice thickness of 0.625 mm were generated directly from the original raw projection data using the scanner reconstruction algorithm. No post-acquisition resampling or interpolation of the 2.5 mm images was performed before volumetric segmentation. The 0.625 mm reconstructed DICOM datasets were transferred to a dedicated volumetric imaging workstation (Vitrea v6.3, Toshiba Medical Systems, Tokyo, Japan) and used for all volumetric measurements.

### 2.4. Volumetric Segmentation and Three-Dimensional Reconstruction

**Figure 1:**
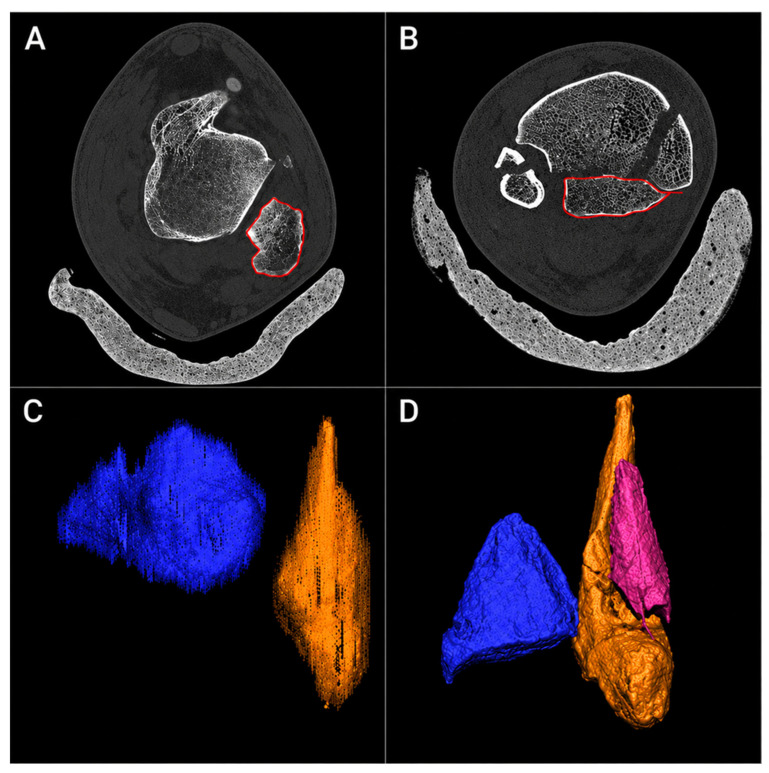
CT-based manual volumetric segmentation and three-dimensional reconstruction of malleolar fracture fragments. (**A**) Axial thin-slice CT image demonstrating manual contour-based segmentation of the lateral malleolar fragment using a free-hand region of interest. (**B**) Axial thin-slice CT image demonstrating manual contour-based segmentation of the posterior malleolar fragment. The red outlines in panels A and B indicate the manually drawn contour boundaries used for segmentation. (**C**) Isolated three-dimensional reconstructed fracture fragments after volumetric segmentation, showing the posterior malleolar fragment in blue and the lateral malleolar fragment in orange. (**D**) Three-dimensional reconstructed CT fragment model demonstrating a multifragmentary configuration, including the posterior malleolar fragment in blue, the primary lateral malleolar fragment in orange, and a secondary lateral malleolar fragment in pink. These reconstructed models were used to support volumetric assessment and morphological review of the fracture fragments.

All volumetric analyses were performed using a standardized manual segmentation workflow. Before segmentation, each CT dataset underwent standardized multiplanar reorientation to minimize variation related to spatial orientation. Reorientation was performed using three orthogonal planes to align the ankle consistently before fragment delineation. Posterior and lateral malleolar fracture fragments were evaluated on 0.625 mm thin-slice axial CT images using manual contour-based slice-by-slice segmentation (Figure 1A,B). Segmentation was performed on the Vitrea workstation using the software’s manual free-hand contour tools. Automated threshold-based segmentation and Hounsfield unit-based automated segmentation methods were not used. This decision was based on both anatomical and technical considerations. The irregular morphology of fracture fragments, their variable degree of displacement, and their close anatomical relationship with adjacent osseous structures could compromise accurate automated boundary detection and result in unintended inclusion of neighboring bone. Therefore, manual slice-by-slice contouring was selected to ensure precise anatomical delineation of individual fracture fragments. Fragment boundaries were manually delineated on consecutive axial slices according to cortical disruption lines, fragment continuity, fracture line configuration, and the degree of anatomical separation from adjacent osseous structures. For lateral malleolar volumetry, all fracture components originating from the distal fibula and anatomically separated by fracture lines were included in the segmentation process. In multifragmentary fracture patterns, each identifiable lateral malleolar fragment was segmented separately, and the total lateral malleolar volume was calculated as the sum of all segmented lateral malleolar components. Thus, volumetric measurements represented the entire fractured lateral malleolar construct rather than only the largest fragment. Small secondary lateral malleolar fragments were not excluded if they could be clearly identified as part of the distal fibular fracture complex. Lateral and posterior malleolar fragments were included in the segmentation when they could be clearly distinguished from the main tibial and fibular osseous structures. After manual contouring of all relevant slices, the software automatically calculated fragment volumes and recorded them in cubic centimeters (cm^3^). Following volumetric segmentation, three-dimensional surface reconstructions were generated using the workstation’s volumetric rendering and bone-removal modules. Posterior and lateral malleolar fragments were isolated from surrounding osseous structures and visualized separately to support volumetric assessment and morphological review (Figure 1C,D). The reconstructed three-dimensional models also enabled visualization of multifragmentary configurations, including cases with more than one lateral malleolar fragment component. The average segmentation time was approximately 15 min per patient.

### 2.5. Observers and Measurement Protocol

All measurements were independently performed by a radiologist with 10 years of experience in musculoskeletal imaging and an orthopedic surgeon with 5 years of experience in ankle trauma imaging. Before formal measurements, both observers underwent a standardized calibration session regarding software use and the segmentation workflow. During this session, multiplanar reorientation, manual contour-based slice-by-slice segmentation, definition of fragment boundaries, handling of multifragmentary fracture components, and three-dimensional reconstruction procedures were standardized using pilot cases that were not included in the final analysis. All DICOM datasets were anonymized before analysis and presented to the observers in a randomized order. The observers were blinded to patients’ clinical information, treatment details, and each other’s measurement results. Each observer performed all segmentation procedures independently and from the beginning. Previously generated segmentation contours, region-of-interest definitions, and measurement outputs were not accessible to the other observer. To evaluate measurement repeatability, all segmentation procedures were repeated by the same observers at a separate time point. A two-week interval was allowed between the first and second measurement sessions. Before the second session, cases were re-randomized, and access to previous segmentation contours, measurement results, and saved region-of-interest data was restricted. This approach was used to reduce recall bias and minimize the potential influence of previous measurements.

### 2.6. Intra-Observer and Inter-Observer Reliability and Geometric Agreement Analysis

Intraobserver reproducibility was assessed by comparing the first and second measurements of the same observer. Intraobserver analyses were performed separately for the orthopedic surgeon and the radiologist. Interobserver reproducibility was assessed by comparing the first measurements of the orthopedic surgeon with the first measurements of the radiologist. Because all measurements consisted of continuous volumetric variables, reproducibility was evaluated using a two-way random-effects, absolute-agreement, single-measure intraclass correlation coefficient model [ICC(A,1)] [6]. An absolute-agreement model was selected because the study aimed to determine whether volumetric measurements could be used interchangeably between repeated measurements and between observers. ICC values were reported with 95% confidence intervals. In addition, Bland–Altman analyses were performed to assess intraobserver and interobserver agreement. Mean bias and 95% limits of agreement were calculated for each comparison. To further evaluate segmentation reproducibility beyond volume-based agreement, geometric agreement between observer-derived segmentation masks was additionally assessed using the Dice similarity coefficient (DSC). DSC was calculated for both posterior and lateral malleolar fracture fragment segmentations in intraobserver and interobserver comparisons. DSC values range from 0 to 1, with higher values indicating greater spatial overlap between segmentation masks. Values above 0.90 were considered indicative of excellent geometric agreement. Mean DSC values with standard deviations and 95% confidence intervals were calculated for all comparisons.

### 2.7. Statistical Analysis

All statistical analyses were performed using IBM SPSS Statistics version 26.0 (IBM Corp., Armonk, NY, USA). Continuous variables were presented as mean ± standard deviation or median with interquartile range, as appropriate. Categorical variables were presented as frequencies and percentages. The distribution of continuous variables was assessed using the Shapiro–Wilk test and visual inspection of histograms. The relationship between posterior and lateral malleolar fragment volumes was evaluated using Spearman’s rank correlation analysis. A non-parametric correlation method was selected because the volumetric variables did not show a normal distribution. The correlation coefficient (rho) and corresponding *p* value were reported. Multivariable linear regression analysis was performed to determine whether the association between lateral and posterior malleolar fragment volumes remained independent of age, sex, and body mass index. Posterior malleolar fragment volume was entered as the dependent variable, whereas lateral malleolar fragment volume, age, sex, and body mass index were entered as independent variables. Sex was coded as a binary categorical variable, with male coded as 0 and female coded as 1. Multicollinearity was assessed using variance inflation factor values. Regression model assumptions were evaluated by visual inspection of residual distributions and by assessment of residual normality, linearity, homoscedasticity, and influential outliers. Although the volumetric variables showed non-normal distributions, non-transformed values were retained because regression assumptions were not substantially violated and preservation of the original measurement scale improved interpretability. A *p* value of <0.05 was considered statistically significant for all analyses.

## 3. Results

### 3.1. Patient and Imaging Characteristics

A total of 71 patients with SER-type trimalleolar ankle fractures were included in the final analysis. The mean age of the study population was 51.5 ± 16.8 years, and 47 patients (66.2%) were female. Right-sided injuries were more frequent than left-sided injuries (63.4% vs. 36.6%). The median lateral malleolar fragment volume was 8.63 cm^3^ (IQR, 7.18–10.71), whereas the median posterior malleolar fragment volume was 2.64 cm^3^ (IQR, 1.88–4.24) (Table 1).

### 3.2. Relationship Between Lateral and Posterior Fragment Volumes

**Figure 2:**
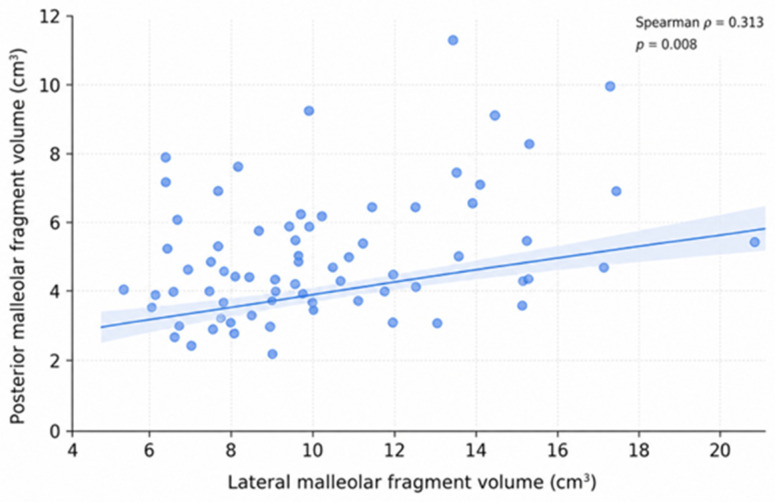
Scatter plot showing the relationship between lateral and posterior malleolar fragment volumes in SER-type trimalleolar ankle fractures. Each dot represents an individual patient, the solid line represents the fitted regression line, and the shaded area indicates the 95% confidence interval. A weak but statistically significant positive correlation was observed between the two volumetric measurements (Spearman rho = 0.313, *p* = 0.008). SER, supination–external rotation.

Posterior and lateral malleolar fragment volumes demonstrated non-normal distributions according to Shapiro–Wilk testing (*p* < 0.001 for both). Therefore, non-parametric statistical methods were used for volume-based correlation analysis. Spearman correlation analysis demonstrated a weak but statistically significant positive correlation between lateral and posterior malleolar fragment volumes (rho = 0.313, *p* = 0.008). Patients with larger lateral malleolar fragments tended to have larger posterior malleolar fragment volumes (Figure 2).

### 3.3. Multivariable Linear Regression Analysis

Multivariable linear regression analysis was performed to evaluate whether the association between lateral and posterior malleolar fragment volumes remained independent of age, sex, and body mass index. Lateral malleolar fragment volume remained independently associated with posterior malleolar fragment volume after adjustment for covariates (B = 0.316, standardized β = 0.39, *p* = 0.002). Age showed a weak negative association with posterior malleolar fragment volume (B = −0.030, standardized β = −0.24, *p* = 0.044). Sex and body mass index were not independently associated with posterior malleolar fragment volume. The overall regression model explained a limited but statistically significant proportion of the variance in posterior malleolar fragment volume (R^2^ = 0.185; adjusted R^2^ = 0.135; model *p* = 0.008). No significant multicollinearity was identified (all VIF values < 5) (Table 2).

### 3.4. Intraobserver and Interobserver Reliability

**Figure 3:**
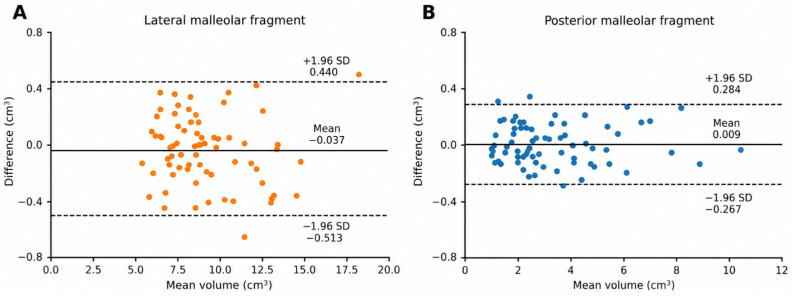
Bland–Altman plots demonstrating interobserver agreement for CT-based volumetric measurements. (**A**) Bland–Altman analysis for lateral malleolar fragment volume measurements obtained by the orthopedist and radiologist. (**B**) Bland–Altman analysis for posterior malleolar fragment volume measurements obtained by the orthopedist and radiologist. The solid horizontal line represents the mean bias, and the dashed horizontal lines indicate the 95% limits of agreement.

Intraobserver and interobserver reproducibility analyses demonstrated excellent agreement for both lateral and posterior malleolar fragment volume measurements. All ICC(A,1) values ranged from 0.996 to 0.999. Bland–Altman analyses demonstrated low mean bias and narrow 95% limits of agreement across all comparisons, supporting the high reproducibility of the CT-based volumetric segmentation workflow (Table 3). Representative Bland–Altman plots for interobserver agreement are presented in Figure 3. Geometric agreement analysis demonstrated excellent spatial overlap for both intraobserver and interobserver comparisons. Dice similarity coefficients ranged from 0.93 to 0.96 across all analyses. Interobserver DSC values were 0.94 ± 0.04 for lateral malleolar fragments and 0.93 ± 0.05 for posterior malleolar fragments, indicating excellent geometric agreement between observer-derived segmentation masks. Detailed DSC results are presented in Table 4.

## 4. Discussion

In this retrospective CT-based volumetric study, lateral and posterior malleolar fragment volumes demonstrated a weak but statistically significant positive association in a homogeneous cohort of Lauge–Hansen supination–external rotation (SER)-type trimalleolar ankle fractures. More importantly, the standardized manual three-dimensional volumetric segmentation workflow showed excellent intraobserver and interobserver reproducibility. ICC(A,1) values ranged from 0.996 to 0.999 across all volumetric measurements, and Bland–Altman analyses demonstrated low mean bias with narrow limits of agreement. These findings suggest that CT-based manual volumetric segmentation can provide highly reproducible quantitative measurements of lateral and posterior malleolar fracture fragments when performed using a standardized workflow.

Posterior malleolar fractures are commonly evaluated using fragment percentage, axial linear measurements, and morphology-based classification systems [1,2]. Although these approaches are clinically practical, they may not fully characterize the spatial complexity of posterior malleolar fracture morphology. Posterior malleolar fragments frequently demonstrate oblique orientation, irregular fracture surfaces, articular extension, and multifragmentary configurations. Therefore, planar measurements may provide only a partial representation of the true three-dimensional fracture geometry. Recent CT-based studies have shown that posterior malleolar morphology is heterogeneous not only in terms of fragment size but also with respect to fracture line configuration, medial extension, fracture height, and articular morphology [8,9,10]. These observations support the need for more standardized and quantitative imaging approaches in the assessment of malleolar fracture morphology.

The present study contributes to this field by evaluating lateral and posterior malleolar fragment volumes simultaneously rather than assessing the posterior malleolus as an isolated component. To the best of our knowledge, only limited evidence is available regarding the three-dimensional volumetric relationship between these two fracture components in SER-type trimalleolar ankle fractures. The SER pattern provides a relatively homogeneous injury model because the lateral and posterior malleolar components arise within a shared rotational injury mechanism [7]. Within this framework, volumetric analysis may offer additional quantitative information beyond conventional classification systems or fragment-size estimates.

The observed association between lateral and posterior malleolar fragment volumes was statistically significant but weak. This finding should not be interpreted as indicating a simple direct relationship between the two fragments. Rather, it suggests that although a measurable volumetric association may exist, posterior malleolar morphology is likely influenced by multiple biomechanical and anatomical factors. In SER-type fractures, fragment morphology may be affected by the degree of talar rotation, direction of load transfer, foot position at the time of injury, bone quality, posterior inferior tibiofibular ligament tension, and the pattern of articular impaction [7]. Therefore, the limited strength of the association is biologically plausible and supports the concept that posterior malleolar morphology cannot be explained solely by the size of the lateral malleolar fragment.

The multivariable linear regression analysis further supported this interpretation. Lateral malleolar fragment volume remained independently associated with posterior malleolar fragment volume after adjustment for age, sex, and body mass index. However, the model explained only a limited proportion of the variance in posterior fragment volume. This indicates that lateral fragment volume may contribute to posterior fragment morphology, but other unmeasured anatomical, biomechanical, and injury-related factors are likely involved. The weak negative association between age and posterior fragment volume observed in the regression model is difficult to interpret clinically within the scope of this study and should be considered exploratory.

A key strength of this study is the excellent reproducibility of the CT-based volumetric segmentation workflow. For any quantitative imaging method to be useful in clinical or research settings, the measurement process must be repeatable, reproducible, and sufficiently standardized [4,5,6]. This requirement is particularly important for manual segmentation, where observer-dependent contour delineation may theoretically introduce measurement variability. In the present study, high ICC values and low Bland–Altman bias values were observed for both intraobserver and interobserver comparisons. Previous studies have reported variable intraobserver and interobserver agreement for morphology-based posterior malleolar fracture classification systems [11]. In this context, the reproducibility of the applied manual CT-based volumetric workflow may represent a methodological advantage for quantitative fracture morphology assessment. Several factors may have contributed to the high reproducibility observed in this study, including thin-slice CT reconstruction, standardized multiplanar reorientation, predefined fragment boundary criteria, observer calibration before formal measurement, independent segmentation, case randomization, and blinding to previous measurements and clinical data. A further strength of the revised analysis is the addition of geometric overlap assessment using the Dice similarity coefficient. Volume-based agreement alone may not fully demonstrate segmentation reproducibility, because different segmentation masks may theoretically yield similar volumes. In the present study, however, DSC values ranging from 0.93 to 0.96 indicated excellent spatial overlap between observer-derived segmentations. Therefore, the reproducibility of the workflow was supported not only by ICC and Bland–Altman analyses but also by direct geometric agreement of the segmentation masks.

From an imaging perspective, CT-based volumetry may serve as a complementary quantitative approach for characterizing fracture morphology. The clinical significance of CT-based volumetric assessment should be interpreted within the context of its role as a quantitative imaging tool rather than as a direct determinant of surgical decision-making. Contemporary management of trimalleolar ankle fractures increasingly relies on detailed characterization of posterior malleolar morphology, articular involvement, syndesmotic stability, and fracture pattern complexity. In particular, these morphological characteristics play a central role in determining indications for posterior malleolar fixation and selecting the surgical approach [1,12]. However, many currently used imaging parameters are based on two-dimensional measurements or qualitative morphological assessments. Volumetric analysis may provide a complementary quantitative approach that reflects the true three-dimensional configuration of fracture fragments and may contribute to a more objective characterization of fracture morphology. The present study demonstrated that CT-based volumetric measurements of posterior and lateral malleolar fracture fragments can be obtained with robust intraobserver and interobserver reproducibility. This finding is clinically relevant because any imaging parameter intended for use in fracture classification systems, surgical planning algorithms, prognostic models, or outcome prediction must first demonstrate that it can be measured reliably and consistently [6,13]. The high ICC and DSC values observed in the present study support the methodological robustness and reproducibility of the proposed volumetric assessment workflow. Only a weak association was identified between posterior and lateral malleolar fragment volumes. This finding suggests that the volumetric relationship between these two fracture components is limited within SER-type trimalleolar ankle fractures. However, the biomechanical and clinical implications of this observation cannot be determined from the present study and should therefore be interpreted cautiously. Rather than establishing a clinically actionable relationship between posterior and lateral malleolar fragment volumes, the current findings should be considered primarily descriptive. Accordingly, the principal contribution of this study is not the immediate application of volumetric measurements to treatment decisions, but rather the establishment of a reliable and reproducible three-dimensional quantitative framework for fracture assessment. Future studies should investigate whether volumetric parameters are associated with posterior malleolar fixation requirements, syndesmotic instability, reduction quality, postoperative joint congruity, and long-term functional outcomes, and whether they provide incremental value beyond existing CT-based classification systems and conventional morphologic measurements.

Unlike categorical classification systems, volumetric assessment generates continuous numerical data and may allow more detailed comparison of fracture patterns in clinical research [4,5,6]. However, volumetric assessment should not be interpreted as a replacement for established clinical, radiographic, or intraoperative evaluation. Important features such as articular impaction, fragment displacement, incisura involvement, syndesmotic stability, soft-tissue injury, and fixation feasibility remain essential for surgical decision-making. Therefore, the present findings support CT-based volumetry as a reproducible research and imaging tool, while its direct clinical utility requires further validation.

This study has several limitations. First, it was retrospective and conducted at a single center. Second, the study population was limited to SER-type trimalleolar ankle fractures to reduce morphological heterogeneity; therefore, the findings may not be generalizable to other ankle fracture mechanisms, such as pronation–external rotation or pronation–abduction injuries. Third, clinical outcomes, functional scores, surgical fixation strategies, and postoperative radiographic outcomes were not evaluated. Consequently, this study cannot determine whether volumetric measurements are associated with surgical decision-making or patient prognosis. Fourth, segmentation was performed manually, which is relatively time-consuming and may limit immediate clinical applicability. Another limitation is that all volumetric segmentations were performed using a single commercial software platform (Vitrea). Although the segmentation workflow relied primarily on manual contour delineation rather than software-specific automated algorithms, reproducibility across alternative commercial or open-source segmentation platforms was not evaluated. Future studies should investigate whether similar reproducibility can be achieved using different segmentation software environments. Fifth, volumetric assessment does not fully capture other clinically relevant morphological features, including marginal articular impaction, osteochondral fragments, displacement, comminution pattern, incisura involvement, and syndesmotic instability. These morphologic characteristics were not systematically evaluated because the primary objective of the present study was volumetric assessment and reproducibility rather than comprehensive morphologic characterization of fracture patterns. Finally, the sample size was limited, and external validation was not performed. Larger multicenter studies are needed to confirm the reproducibility of this workflow and to evaluate whether CT-based volumetric parameters have prognostic or surgical relevance.

In summary, this study demonstrated that manual CT-based three-dimensional volumetric segmentation of lateral and posterior malleolar fragments can be performed with excellent intraobserver and interobserver reproducibility in SER-type trimalleolar ankle fractures. The weak but independent association between lateral and posterior malleolar fragment volumes suggests a measurable, although limited, three-dimensional relationship between these fracture components. Future prospective studies should investigate whether CT-based volumetric parameters improve fracture characterization, surgical planning, or outcome prediction when combined with established morphological and clinical assessment methods.

## 5. Conclusions

This retrospective CT-based volumetric study demonstrated a weak but independent positive association between lateral and posterior malleolar fragment volumes in SER-type trimalleolar ankle fractures. The standardized manual three-dimensional volumetric segmentation workflow showed excellent intraobserver and interobserver reproducibility. These findings suggest that CT-based volumetry may provide a reproducible complementary quantitative imaging approach for assessing malleolar fracture morphology. However, the clinical relevance of this method for surgical decision-making, fixation planning, and patient outcomes should be evaluated in larger prospective studies.

## Figures and Tables

**Table 1:** Demographic, Clinical, and Volumetric Characteristics of the Study Population.

Variable	Value
Number of patients	71
Age (years)	51.5 ± 16.8
Male sex, *n* (%)	24 (33.8%)
Female sex, *n* (%)	47 (66.2%)
Height (m)	1.66 ± 0.10
Weight (kg)	77.8 ± 14.8
BMI (kg/m^2^)	28.1 ± 4.4
Right-sided injury, *n* (%)	45 (63.4%)
Left-sided injury, *n* (%)	26 (36.6%)
Lateral fragment volume (cm^3^), median (IQR)	8.63 (7.18–10.71)
Posterior fragment volume (cm^3^), median (IQR)	2.64 (1.88–4.24)

BMI, body mass index; IQR, interquartile range. Continuous variables are presented as mean ± standard deviation or median (interquartile range), depending on data distribution.

**Table 2:** Multivariable linear regression analysis for factors associated with posterior malleolar fragment volume.

Variable	B	Standardized β	95% CI	*p*	VIF
Lateral fragment volume	0.316	0.39	0.12 to 0.51	0.002	1.08
Age	−0.030	−0.24	−0.06 to −0.001	0.044	1.05
Sex	−0.954	−0.18	−2.10 to 0.19	0.100	1.03
BMI	0.062	0.14	−0.04 to 0.17	0.241	1.07

Model statistics: R^2^ = 0.185; Adjusted R^2^ = 0.135; model *p* = 0.008. B, unstandardized regression coefficient; BMI, body mass index; CI, confidence interval; VIF, variance inflation factor.

**Table 3:** Intraobserver and Interobserver Reliability of CT-Based Volumetric Measurements.

Measurement	Analysis	Comparison	*n*	ICC(A,1)	95% CI	Mean Difference	95% LoA	Interpretation
Lateral malleolar volume	Intra-observer	Orthopedist T2 vs. T1	71	0.998	0.996–0.999	−0.010	−0.348 to 0.329	Excellent
Lateral malleolar volume	Intra-observer	Radiologist T2 vs. T1	71	0.999	0.998–0.999	−0.005	−0.269 to 0.259	Excellent
Posterior malleolar volume	Intra-observer	Orthopedist T2 vs. T1	71	0.999	0.998–0.999	−0.007	−0.199 to 0.184	Excellent
Posterior malleolar volume	Intra-observer	Radiologist T2 vs. T1	71	0.999	0.998–0.999	0.010	−0.177 to 0.197	Excellent
Lateral malleolar volume	Inter-observer	Radiologist T1 vs. Orthopedist T1	71	0.996	0.993–0.997	−0.037	−0.513 to 0.440	Excellent
Posterior malleolar volume	Inter-observer	Radiologist T1 vs. Orthopedist T1	71	0.998	0.996–0.998	0.009	−0.267 to 0.284	Excellent

ICC(A,1), intraclass correlation coefficient for absolute agreement; CI, confidence interval; LoA, limits of agreement; T1, first measurement; T2, second measurement. Bland–Altman analyses were used to evaluate agreement between measurements.

**Table 4:** Geometric Agreement Analysis Using Dice Similarity Coefficients.

Measurement	Analysis	Comparison	*n*	DSC, Mean ± SD	95% CI	Interpretation
Lateral malleolar fragment	Intraobserver	Orthopedist T2 vs. T1	71	0.95 ± 0.03	0.94–0.96	Excellent
Lateral malleolar fragment	Intraobserver	Radiologist T2 vs. T1	71	0.96 ± 0.02	0.95–0.96	Excellent
Posterior malleolar fragment	Intraobserver	Orthopedist T2 vs. T1	71	0.94 ± 0.04	0.93–0.95	Excellent
Posterior malleolar fragment	Intraobserver	Radiologist T2 vs. T1	71	0.95 ± 0.03	0.94–0.96	Excellent
Lateral malleolar fragment	Interobserver	Radiologist T1 vs. Orthopedist T1	71	0.94 ± 0.04	0.93–0.95	Excellent
Posterior malleolar fragment	Interobserver	Radiologist T1 vs. Orthopedist T1	71	0.93 ± 0.05	0.92–0.94	Excellent

DSC, Dice similarity coefficient; SD, standard deviation; CI, confidence interval; T1, first segmentation session; T2, second segmentation session. DSC values range from 0 to 1, with higher values indicating greater spatial overlap between segmentation masks.

## Data Availability

The data presented in this study are available from the corresponding author upon reasonable request. The data are not publicly available due to privacy and ethical restrictions.
